# Higher prefrontal activity based on short-term neurofeedback training can prevent working memory decline in acute stroke

**DOI:** 10.3389/fnsys.2023.1130272

**Published:** 2023-06-14

**Authors:** Masayuki Tetsuka, Takeshi Sakurada, Mayuko Matsumoto, Takeshi Nakajima, Mitsuya Morita, Shigeru Fujimoto, Kensuke Kawai

**Affiliations:** ^1^Department of Neurosurgery, Jichi Medical University, Tochigi, Japan; ^2^Faculty of Science and Technology, Seikei University, Tokyo, Japan; ^3^Functional Brain Science Laboratory, Center for Development of Advanced Medical Technology, Jichi Medical University, Tochigi, Japan; ^4^College of Systems Engineering and Science, Shibaura Institute of Technology, Saitama, Japan; ^5^Rehabilitation Center, Jichi Medical University Hospital, Tochigi, Japan; ^6^Division of Neurology, Department of Medicine, Jichi Medical University, Tochigi, Japan

**Keywords:** acute stroke, functional near-infrared spectroscopy, neurofeedback, working memory, prefrontal cortex

## Abstract

This study aimed to clarify whether short-term neurofeedback training during the acute stroke phase led to prefrontal activity self-regulation, providing positive efficacy to working memory. A total of 30 patients with acute stroke performed functional near-infrared spectroscopy-based neurofeedback training for a day to increase their prefrontal activity. A randomized, Sham-controlled, double-blind study protocol was used comparing working memory ability before and after neurofeedback training. Working memory was evaluated using a target-searching task requiring spatial information retention. A decline in spatial working memory performance post-intervention was prevented in patients who displayed a higher task-related right prefrontal activity during neurofeedback training compared with the baseline. Neurofeedback training efficacy was not associated with the patient’s clinical background such as Fugl–Meyer Assessment score and time since stroke. These findings demonstrated that even short-term neurofeedback training can strengthen prefrontal activity and help maintain cognitive ability in acute stroke patients, at least immediately after training. However, further studies investigating the influence of individual patient clinical background, especially cognitive impairment, on neurofeedback training is needed. Current findings provide an encouraging option for clinicians to design neurorehabilitation programs, including neurofeedback protocols, for acute stroke patients.

## 1. Introduction

Self-regulation of neural activity patterns using neurofeedback improves specific brain functions in healthy individuals (Escolano et al., 2011; Wang and Hsieh, 2013; Rozengurt et al., 2016) and clinical populations (Mihara and Miyai, 2016; Wang et al., 2018). Indeed, neurofeedback-based neurorehabilitation has been reported effective for modulating brain activity in various conditions, such as stroke, epilepsy, attention deficit hyperactivity disorder, and schizophrenia (Papo, 2019). Regarding the type of neuroimaging equipment, recently, the number of neurofeedback studies using functional near-infrared spectroscopy (fNIRS) has increased (Ehlis et al., 2018; Kohl et al., 2020). Notably, previous studies focused on the prefrontal cortex as a region of interest. For instance, neurofeedback modulation of the right dorsolateral prefrontal cortex (DLPFC) can improve emotional regulation (Yu et al., 2021), bilateral DLPFC activity decreases social anxiety disorder (Kimmig et al., 2019), bilateral frontal pole cortex activity is associated with metacognitive abilities (Kinoshita et al., 2016), asymmetric bilateral DLPFC activity can contribute to controlling mental disposition (Aranyi et al., 2016), and DLPFC also improves executive function performance (Barth et al., 2016; Hosseini et al., 2016). Furthermore, another study targeting other areas than the prefrontal cortex showed that higher supplementary motor area activity affects postural stability (Fujimoto et al., 2017).

Functional near-infrared spectroscopy-based neurofeedback trainings have been efficiently used in patients after stroke to modulate motor- and cognitive-related processes. For instance, utilizing fNIRS-based neurofeedback to target the premotor area while conducting motor imagery of a paretic hand’s movements resulted in better recovery of finger motor function in patients with hemiplegic stroke. Specifically, during neurofeedback training, changes in motor imagery-related premotor activity were significantly correlated with functional recovery (Mihara et al., 2013). A more recent study has proposed a neurofeedback system combining fMRI and fNIRS protocols to facilitate motor learning in patients with stroke (Rieke et al., 2020). However, regardless of the imaging equipment, most neurofeedback studies targeted patients during the stroke subacute stage occurring from 15 days to 6 months post-stroke, or during the chronic stage, i.e., beyond 6 months post-stroke (Mihara and Miyai, 2016; Renton et al., 2017; Wang et al., 2018). One of the few reports on this issue concerns four patients with acute stroke with spatial neglect (mean post-stroke delay, 10.5 days). These patients successfully acquired lower alpha power in the posterior cortex during electroencephalogram (EEG)-based neurofeedback training, and showed behavioral improvements in clinical visuospatial tasks (Saj et al., 2018). However, the data on acute patients are not sufficient, and whether neurofeedback training is an effective approach during the stroke acute phase remains to be determined.

Individual working memory (WM) ability for spatial information in a healthy population was recently reported to be facilitated by short-term fNIRS-based neurofeedback training for the prefrontal cortex. Furthermore, individuals who were proficient at holding somatosensory information were demonstrated to show higher training efficacy than those who were proficient at holding visual information (Sakurada et al., 2022). These findings imply that short-term neurofeedback training is effective even in acute stroke patients if they retain the cognitive capacity to process information of a particular sensory modality, especially tactile or somatosensory information.

This study aims to clarify whether short-term neurofeedback training during acute stroke successfully results in prefrontal activity self-regulation and positive benefits for WM. To address these aims, the present neurofeedback protocol delivered feedback on the bilateral DLPFC and frontopolar cortex (FPC) activities, which play a crucial role in WM (Owen et al., 2005; Pleger et al., 2006; Slotnick and Moo, 2006; Kaas et al., 2007; Giglia et al., 2014) and are one of the neurological bases underlying the individual aspects of WM reflecting sensory processing ability (Matsumoto et al., 2020).

## 2. Materials and methods

### 2.1. Participants

**FIGURE 1:**
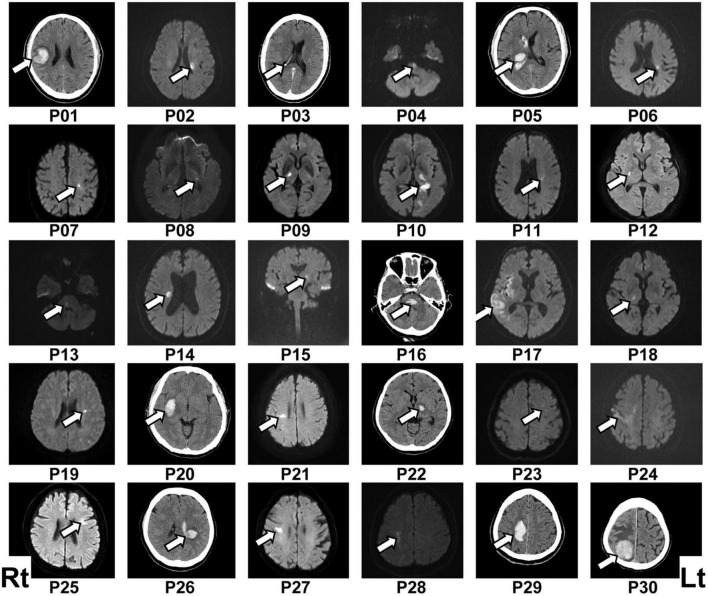
Lesion location in each patient. The patient number (Pxx) was given according to the order of participation. Lesions were located mainly by diffusion-weighted imaging following ischemic stroke (P02, P04, P06–P15, P17–P19, P21, P23–P25, P27, and P28) or computed tomography (CT) scan following hemorrhagic stroke (P01, P05, P16, P20, P22, P26, P29, and P30). The lesion location in the ischemic stroke patient P03 was determined via CT scan because of a contraindication for using diffusion-weighted imaging. The left (Lt) side of the figure represents the right (Rt) side of the brain.

Two hundred seventy-eight patients with acute stroke were enrolled from the Department of Neurosurgery and Division of Neurology, Department of Internal Medicine, Jichi Medical University. Patients with subarachnoid hemorrhage were excluded, as were patients with upper limb movement deficits unrelated to stroke as well as those with aphasia, dysarthria, or visual field loss. Because the experimental task required patients to move their affected hands while holding a digitizing pen, stroke patients with severe paralysis (i.e., manual muscle test grading less than 3) and with sensory loss in the upper limb were also excluded. Because the mini-mental state examination (MMSE) (Folstein et al., 1975) is one of the most used tests for evaluating the cognitive function, patients with MMSE scores below 24 were also excluded. After exclusion, the remaining 36 patients were randomly allocated into the experimental groups. Finally, 30 patients with acute hemiparetic stroke were included in the final analysis. For the 30 patients, we used the Fugl–Meyer Assessment of the upper extremity motor score (FMA motor score) to rate motor recovery after stroke (Fugl-Meyer et al., 1975). The patient information is summarized in Table 1 and Figure 1. This study was conducted following the Declaration of Helsinki and approved by the Institutional Review Board at Jichi Medical University and all patients provided written informed consent before participation. See Supplementary Figure 1 for CONSORT flow diagram.

**TABLE 1:** Participant information.

Variable	Overall (mean ± SD)	Real group	Sham group	*p*-value
Number of patients	30	20	10	–
Age (years)	62.6 ± 12.8	61.8 ± 14.4	64.1 ± 9.5	0.79
Gender	14F/16M	10F/10M	4F/6M	–
Handedness	0Lt/30Rt	0Lt/20Rt	0Lt/10Rt	–
Affected side	16Lt/14Rt	10Lt/10Rt	6Lt/4Rt	–
Time since stroke (days)	5.7 ± 3.3	5.7 ± 3.1	5.7 ± 3.9	0.89
**Stroke type**				
Infarction	22	14	8	–
Hemorrhage	8	6	2	–
**Stroke lesion**				
Subcortical cerebrum	7	4	3	–
Corona radiata	11	8	3	–
Putamen	2	2	0	–
Thalamus	5	2	3	–
Internal capsule	2	1	1	–
Pons	3	3	0	–
MMSE (/30)	27.8 ± 1.6	27.9 ± 1.7	27.7 ± 1.4	0.70
FMA motor score (/66)	55.0 ± 3.1	54.7 ± 3.5	55.8 ± 2.0	0.36
**Manual muscle test**				
5−	21	12	9	–
4 +	7	7	0	–
4	2	1	1	–

Age, time since stroke, MMSE and FMA motor score were compared between experimental groups by the Wilcoxon rank sum test. SD, standard deviation; Lt, left; Rt, right; F, female; M, male; MMSE, mini-mental state examination; FMA, Fugl–Meyer assessment.

Note that their attending physicians suspected cognitive impairment in 9/30 final patients. For the suspected nine patients, this study also referred to other cognitive function test results performed before the experimental participation. Specifically, the Frontal Assessment Battery (Dubois et al., 2000), the Behavioral Inattention Test (Wilson et al., 1987), the Trail Making Test (Corrigan and Hinkeldey, 1987), or the revised version of Hasegawa’s Dementia Scale (Imai and Hasegawa, 1994) was administered to the patients with suspected cognitive dysfunction. These evaluations found that three patients had mild cognitive dysfunction (see Supplementary Table 1 for detailed data regarding each patient); however, the patients with mild cognitive dysfunction underwent the same experimental protocol as that used in the patients without cognitive dysfunction.

The final 30 patients performed an fNIRS-based neurofeedback training session to regulate bilateral prefrontal activity as well as a behavioral task to assess WM ability before and after neurofeedback training. These experimental tasks (Sections “2.2. Regulation of the prefrontal activity by fNIRS-based neurofeedback training” and “2.3. Target-searching task for evaluating spatial WM ability”) are based on our recent research targeting WM in healthy populations (Sakurada et al., 2022), and modified as required for stroke patients. Note that the current tasks were simplified for the purpose of reducing the physical and cognitive burden on the patients. Furthermore, to avoid the effect of spontaneous recovery in the acute phase on the neurofeedback training, each patient completed all experimental tasks including practice trials in a single day, specifically, within a 2.5-h period. We confirmed that each patient correctly understood the procedures of the experimental tasks in the practice trials. All participated patients reported that they did not feel any significant fatigue in a self-assessment during and after the experiment.

### 2.2. Regulation of the prefrontal activity by fNIRS-based neurofeedback training

#### 2.2.1. Experimental setup

**FIGURE 2:**
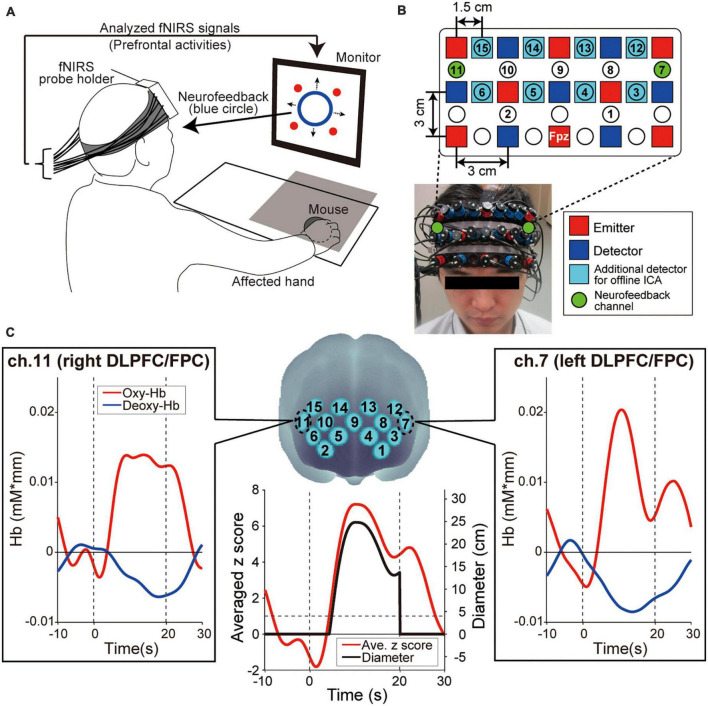
Experimental setup. **(A)** Functional near-infrared spectroscopy (fNIRS)-based neurofeedback training set up. The patient’s hand was hidden by a rack represented by a gray square on the illustration. The four red circles on the monitor indicated the visual stimuli that patients were required to remember. **(B)** Configuration of the fNIRS probe. Probes were placed over the prefrontal area. **(C)** Spatial registration of fNIRS maps onto Montreal Neurological Institute coordinates space. These feedback channels, ch.7 and ch.11, convey signals from the left and right dorsolateral prefrontal cortex (DLPFC) and frontopolar cortex (FPC), respectively. The profiles show typical ones of oxygenated hemoglobin (oxy-Hb), deoxygenated hemoglobin (deoxy-Hb), averaged z score and diameter of the blue circle, respectively. Time zero indicates the onset of the task block. During the task block, the oxy-Hb signals showed a greater response than the deoxy-Hb signals. As values for presenting neurofeedback information, the bilateral oxy-Hb signals were converted to z scores and the z scores were averaged in real-time. Finally, the diameter was calculated from the averaged z score. The size of the blue circle on the display increased only when the z score exceeded 1 and was not displayed during the Rest blocks.

Each patient was seated on a chair facing a monitor (size: H30.5 × W37.7 cm) for visual stimulus presentation and was asked to hold a computer mouse in their affected hand. All visual stimuli presented on the monitor were programmed in MATLAB (MathWorks, Inc., Natick, MA, USA). During the fNIRS-based neurofeedback training task, the affected hand was hidden by a small rack (Figure 2A). To measure the prefrontal activity, we used a multichannel fNIRS system (ETG-7100, Hitachi Medical Corporation, Kashiwa, Japan) with probes arranged to cover the prefrontal area. All fNIRS channel inputs were sampled at 10 Hz. A 3 × 9 multichannel probe holder consisted of eight laser sources emitting at 695 and 830 nm and seven detecting probes alternately arranged at an inter-probe distance of 3 cm. The midpoint of an emitter (red squares in Figure 2B) and detector (blue squares in Figure 2B) was defined as a recording channel location (22 circles in Figure 2B). The probe holder was placed on the scalp with its lowest-row center emitter at the patient’s Fpz position according to the standard international 10–20 system. Regarding the spatial profiling of the recording channels (i.e., correspondence brain area and Brodmann area), we did not measure each recording channel’s 3D position to limit the patients’ experimental time. Therefore, we referred to the spatial profiling of our previous study using the same fNIRS probe settings as the current work (see Table 1 in our previous study; Matsumoto et al., 2020).

#### 2.2.2. Procedure

The neurofeedback training task consisted of six sessions, each comprising five alternating 15-s rest and 20-s task blocks with an additional rest block inserted at the end of each session (i.e., 11 blocks per session). To determine the duration of Rest blocks, it is necessary to consider the time required for the increased activity level obtained in the task blocks to return to baseline levels. Therefore, based on our previous research (Sakurada et al., 2022) and the preliminary results of this study, we estimated the fNIRS response delay to be approximately 10 s and set the rest block length to 15 s, which is slightly longer than the estimated delay. In fact, we confirmed that fNIRS signals returned to the baseline around 10 s in many cases in this study, as shown in Figure 2C.

To calculate the online feedback values originating from brain activities, we performed signal normalization for oxygenated hemoglobin (oxy-Hb) during neurofeedback training. Although we measured oxy-Hb and deoxygenated hemoglobin (deoxy-Hb), we only used the former for neurofeedback because they are more sensitive to changes in cerebral blood flow and present a higher signal-to-noise ratio than deoxy-Hb signals (Toronov et al., 2001; Strangman et al., 2002). As shown in Figure 2C, we observed the same phenomenon regarding the higher oxy-Hb signal changes. Furthermore, since fNIRS signals are relative values, we avoided using the values directly. Instead, the online raw oxy-Hb signals in each channel were normalized to the mean and standard deviation during the 10 s before the beginning of each task block (i.e., z-scoring). Importantly, although no artifact correction was performed in the online fNIRS signals, no artifact was detected in the post-experiment offline analysis.

Based on the z score value during the task blocks, patients received feedback information from the monitor displayed as a blue circle in which the diameter reflected the strength of prefrontal activities in ch.7 and ch.11 (green-colored circles in Figure 2B), corresponding mainly to the bilateral DLPFC (Brodmann area 46) and FPC (Brodmann area 10). The blue circle diameter was determined based on the mean z score value between ch.7 and ch.11. Several studies reported that increasing prefrontal activity is an effective approach to improve cognitive ability in the elderly and in patients with stroke (van Asselen et al., 2006; Jones et al., 2015; Stephens and Berryhill, 2016). Thus, to enhance the bilateral DLPFC/FPC activity, the blue circle size increased when the averaged z score among the two neurofeedback channels increased. More specifically, when the averaged z score was < 1 (i.e., the activities of the feedback channels did not increase), the diameter of the blue circle was 0 and indicated as a dot. When the averaged z score was > 1, the diameter increased linearly with the increasing z score [4 cm/(z score-1), black line in Figure 2C].

To activate spatial WM processing and induce higher prefrontal activities during the task blocks, we required patients to remember the sequential patterns of four visual stimuli presented on the monitor as a cognitive task. Each visual stimulus was shown as a red-filled circle with a diameter of 1 cm. The four visual stimuli were sequentially and individually presented once in random order at predetermined fixed positions (top-left, top-right, bottom-left, and bottom-right position on the monitor) beginning just after the start of each task block, and the order was different for every task block. The presentation duration for each red-filled circle was 0.5 s. Then, immediately after the start of each rest block, patients were required to verbally answer the visual stimuli order of the immediately preceding task block. Concerning the task blocks, patients were also instructed to press the computer mouse at a comfortable constant frequency with their index fingers. This cognitive-motor task was utilized during the task blocks to maintain arousal levels during neurofeedback training by increasing the task difficulty within a reasonable range for the patients. Under our task settings, the participants were instructed to concentrate on both memorizing the sequential pattern of visual stimuli and creating a blue circle as large as possible. Since there were two task targets during neurofeedback training, it is necessary to confirm whether the patients concentrated on them simultaneously, as instructed. Regarding the sequential pattern memorization and the blue circle size, we calculated the correct answer rate, and asked the patients how the circle had changed throughout each session, respectively.

We randomly allocated the patients into a Real group and a Sham group, with the patients and experimental operator being blind to the group assignment. To verify the neurofeedback training efficacy, we assigned a larger number of patients into the Real group (Real/Sham = 20/10). Despite the asymmetric number of patients in the Real and Sham groups, we confirmed the lack of significant differences in age, time since stroke, MMSE, and FMA motor scores between groups, as shown in Table 1. The blue circle diameter on the monitor for the Real group was determined in real-time based on the patients’ own oxy-Hb signals in ch.7 and ch.11. For the Sham group, the blue circle size was based on the prerecorded oxy-Hb signals of another person and was unrelated to the patient’s own cortical activation.

#### 2.2.3. Offline analysis of fNIRS signals

To evaluate how prefrontal activity in each recording channel changed during neurofeedback training, offline analyses were performed on the measured oxy-Hb signals. Specifically, we performed (1) multi-distance independent component analysis to remove the effects of skin blood flow, (2) high-pass filtering to remove drift components, (3) artifact detection and removal, and (4) general linear model analysis to detect task-related cortical activity. Details of each analysis are shown below.

Multi-distance independent component analysis (Note: This analysis is a native function of the fNIRS system used in this study): fNIRS signals reflect hemoglobin changes that originate in cortical tissues because of brain activation and skin blood flow. To eliminate the impact of the skin blood flow on the fNIRS signals, we used eight additional detectors with a short inter-probe distance of 1.5 cm (light blue squares in Figure 2B) and applied multi-distance independent component analysis for the fNIRS analysis (Hirosaka et al., 2004; Morren et al., 2004; Akgül et al., 2006; Kohno et al., 2007; Funane et al., 2014). Signals from recording channels with a 1.5 cm inter-probe distance primarily included skin blood flow signals in shallow tissues. Based on these signals, we discriminated between the effects of cortical tissue and skin blood flow on fNIRS signals. As it was possible to apply multi-distance independent component analysis only to the recording channels around the short detecting probes, the number of available recording channels was reduced to 15 after applying multi-distance independent component analysis (numbered recording channels in Figure 2B).

Remove drift components and artifacts: After eliminating the effect of skin blood flow by the multi-distance independent component analysis, to remove the baseline drift, the individual time courses of the oxy-Hb signal from each channel were high-pass filtered using a cut-off frequency of 0.0143 Hz. Then, to remove blocks with motion-related artifacts (i.e., sharp changes in the oxy-Hb signal), we applied an artifact detection algorithm based on the HOMER2 software (MGH-Martinos Center for Biomedical Imaging).^1^ As we did not detect any block with artifacts, we analyzed all oxy-Hb time course data from this study.

General linear model analysis: Finally, to avoid using the fNIRS relative values directly, we applied general linear model analysis (Friston et al., 1994a,b). General linear model analysis allows detecting task-related hemodynamic changes in the cortex based on fNIRS data (Schroeter et al., 2004; Plichta et al., 2007). To identify neuromodulation in the prefrontal regions related to WM processing of spatial information, we used general linear model analysis with least-squares estimation of the oxy-Hb signals. For the preprocessed oxy-Hb signals, a Gaussian function with a peak time of 6 s and full width at half maximum of 5.4 s was used as a hemodynamic response function to better mimic brain signals. The resulting beta values for each recording channel as estimated by general linear model analysis were then used in the group analysis to evaluate the degree of neuromodulation during neurofeedback training.

Regarding the success criteria of the neurofeedback training used in this study, we focused on the beta values calculated from the general linear model. Specifically, to determine whether neurofeedback training increased task-related activity, we subtracted the individual beta value of the first session from that of the sixth session in each feedback channel. In other words, neurofeedback training was considered successful if this difference was positive.

#### 2.2.4. Statistical analysis

Considering the purpose of neurofeedback training to increase prefrontal activities, Spearman correlation coefficients were calculated to estimate the transition trend in beta values throughout sessions in each group. Then, beta values in the first and sixth sessions were compared for each feedback channel (i.e., ch.7 and ch.11) in each group by Wilcoxon signed-rank tests. Furthermore, to evaluate the differences in neurofeedback training efficacy between the Real and Sham groups, the amount of change in beta value in each feedback channel based on the 1st session (i.e., sixth session minus first session) was compared using Wilcoxon rank-sum tests. The data were analyzed by IBM SPSS Statistics 25. A *p* < 0.05 (two-tailed) was considered significant for all tests.

### 2.3. Target-searching task for evaluating spatial WM ability

#### 2.3.1. Experimental setup

Based on our previous study (Matsumoto et al., 2020; Sakurada et al., 2022), we applied a target-searching task to quantify individual spatial WM abilities. We simplified the setup and content of the experimental tasks for the patients. Each patient was seated on a chair and asked to hold a digitizing pen on a drawing tablet (Intuos4 PTK-1240/K0, Wacom, Japan) with their affected hand. A monitor (size: H30.5 × W37.7 cm) for visual stimulus presentation was placed horizontally 16.5 cm above the tablet. Because the patients’ affected hand was hidden by a cloth and the monitor, they could not directly see it during the experimental tasks. Visual stimuli such as task instructions presented on the monitor were programmed in MATLAB using the Cogent Toolbox. The Cogent Toolbox also recorded the position of the digitizing pen tip with sampling at 60 Hz. A vibration motor was attached to the index fingertip of the affected hand to deliver vibrotactile stimuli.

#### 2.3.2. Procedure

The target-searching task required patients to find four targets on the drawing tablet (searching area: 21.3 × 26.4 cm). The targets were positioned randomly and appeared individually in a predetermined, sequential order. Regarding the stimulus cues presenting the target locations, we introduced an experimental condition based on vibrotactile stimuli. Specifically, during the searching trial, when the tip of the digitizing pen came into a target area on the tablet (diameter: 10 cm), a vibrotactile stimulus was delivered to the index fingertip by the vibration motor to indicate the target location.

In each trial, the patients were first required to move the digitizing pen to the center of the searching area. Then, the background color of the monitor was changed as a start cue and the patients began to search for the first target. When the digitizing-pen entered a target area, the vibrotactile stimulus was presented, and the sensory stimuli continued until the tip of the digitizing-pen moved out of the target area. If the digitizing-pen remained in the target area for 0.7 s, a beep signal informed the patient of successful target detection. Afterward, the patients immediately started to search for the next target. Finally, each trial finished when the patient had found all four targets. Patients were required to find all four targets as quickly as possible. Therefore, they had to retain spatial information, namely, the target locations and appearance orders, in the repeated trials. We expected that patients would gradually show efficient searching as a learning effect (i.e., shorter searching movement trajectory) if they could retain the spatial information of the target in this task.

Patients performed five target-searching trials before and after neurofeedback training (Pre-WM task and Post-WM task), respectively. The four target locations and appearance orders were consistent within five trials in each Pre- and Post-WM task, but new target locations and appearance orders different from the Pre-WM task were applied in the Post-WM task. Thus, patients needed to again retain spatial information in the Post-WM task. To equalize the task difficulty, the total distances among all targets (i.e., the cumulative length of the straight lines connecting all targets from the 1st to the 4th) were the same between Pre- and Post-WM tasks.

#### 2.3.3. Analysis

To quantify the searching task performance reflecting the individual WM ability, we referred to the index applied in our previous studies (Matsumoto et al., 2020; Sakurada et al., 2022). Specifically, as a searching performance index, we calculated the normalized movement distance, which is defined as the total distance traveled by the affected hand divided by the shortest possible distance connecting the four targets by a straight line. We can deduce that the normalized movement distance is strongly related to the individual WM ability because retaining spatial information for hidden targets can optimize the searching movement trajectory on the drawing tablet (i.e., patients can search for the targets in a shorter distance). In this scenario, a greater reduction of the normalized movement distance throughout the trials indicates a greater WM ability to retain the target locations and appearance order.

The first trials in the Pre- and Post-WM tasks were excluded from the task performance analysis because the patients did not know the target locations during the first trial and had to randomly search for the targets without being able to rely on WM information. In other words, the first trial’s search performance was strongly influenced by the random search trajectory taken by each patient, regardless of WM ability. Since it is difficult to remove effects in the first trial unrelated to individual WM ability, the mean normalized movement distance was calculated from the second to the fifth trials in the Pre- and Post-WM tasks. We then compared the mean normalized movement distances between the Pre- and Post-WM tasks to obtain an index of the behavioral outcome reflecting the neurofeedback training efficacy. Specifically, a decreased mean normalized movement distance in the Post-WM task compared with that of the Pre-WM task was considered as an indicator of the performance improvement associated with neurofeedback training. Furthermore, we calculated the proportion of patients showing an improvement in their searching performance in each group.

#### 2.3.4. Statistical analysis

The mean normalized movement distances were analyzed via a two-way repeated-measures analysis of variance (ANOVA) with neurofeedback training groups (Real and Sham) as a between-subject factor and task phase (Pre and Post) as a within-subject factor. We analyzed the proportion of patients with improved performance using z value. Furthermore, we examine the relationship between the degree of neuromodulation during neurofeedback training in the prefrontal activity and the WM ability to hold spatial information. To this aim, we calculated the Spearman correlation coefficient between the inter-subject variance of the beta value change from the first to the sixth session and that of the mean normalized movement distance from the Pre-WM and Post-WM tasks. All data were analyzed using IBM SPSS Statistics 25. A *p* < 0.05 (two-tailed) was considered significant for all tests.

## 3. Results

### 3.1. Prefrontal activity

As mentioned in Section “2.2.2. Procedure,” the patients needed to concentrate on two task targets during neurofeedback training. Regarding the sequential pattern memorization, the correct answer rates of the Real and Sham groups were 95.2% ± 6.7SD and 97.5% ± 4.0SD, respectively, without significant differences between them (*p* = 0.43, Wilcoxon rank-sum test). Regarding the blue circle size, no response showed a trend clearly different from the actual circle size change. Therefore, it can be assumed that both groups performed the training while concentrating on both task targets as instructed.

**FIGURE 3:**
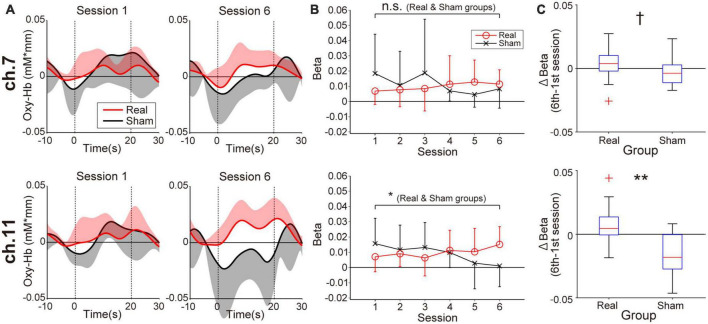
Neuromodulation in the feedback channels (ch.7: upper panels, ch.11: lower panels) during the neurofeedback training task. **(A)** The temporal profiles of oxygenated hemoglobin (oxy-Hb) signals. Red and black lines represent the time courses of the oxy-Hb signals in the Real and Sham groups, respectively. The lighter-colored regions around the time course lines represent the standard deviation. The upper or lower directional standard deviation regions are shown for the profiles of the Real and Sham groups, respectively. In ch.11 of the Real group, clear task-related activity was observed in the final training session. **(B)** Beta value transitions. For ch.11 over the right prefrontal area, oxy-Hb beta values in the Real group increased gradually, whereas they decreased gradually in the Sham group. The beta value change of ch.11 in the Real group was consistent with the neurofeedback training aim, and the amount of change was significant even after Bonferroni correction. Error bars represent the standard deviation. **(C)** Between-group differences in the amount of beta value changes comparing first and sixth sessions. A significant difference was observed only in the right frontal area. ^†^*p* < 0.1, *p < 0.05, **p < 0.01.

Figure 3 shows prefrontal activity patterns based on oxy-Hb signal in feedback channels (Figure 3A) and the corresponding beta value transitions from the first to the sixth session estimated by general linear model analysis (Figure 3B). The trends of beta value transitions in both feedback channels showed marked group differences, with the Real group showing gradually increasing beta values, consistent with the neurofeedback training aims. By contrast, a decreasing trend was observed for the Sham group. The changes in beta values were relatively strong in ch.11 compared to ch.7. Regarding these beta value trends, the correlation coefficients for ch.11 were 0.19 (*p* = 0.036, Real group) and −0.30 (*p* = 0.021, Sham group). The statistical results indicate that the beta values in the Real group tended to increase whereas those in the Sham group tended to decrease over the session. On the other hand, in ch.7, none of the groups showed a marked trend (*r* = 0.17, *p* = 0.056; Real group, *r* = −0.20, *p* = 0.14; Sham group). Despite the weak tendencies of beta value changes, the Real and Sham groups showed an increasing and decreasing trend, similar to ch.11, respectively. Although the current neurofeedback training duration was very short, at least in ch.11 we could observe linear changes in neuromodulation as a training effect. Thus, we compared the results between the first and last sessions, where the differences in brain activity would be the greatest. The Wilcoxon signed-rank tests assessing beta value differences between the first and sixth sessions revealed marked activity changes in ch.11 for the Real group (*p* = 0.0124) and the Sham group (*p* = 0.037). By contrast, no significant change was obtained for ch.7 (*p* = 0.12; Real group, *p* = 0.28; Sham group). As shown in Figure 3C, the Wilcoxon rank-sum tests on the beta value changes between the 6th and 1st sessions revealed a significantly larger change in the Real group than the Sham group, especially in the right frontal area (*p* = 0.0040; ch.11, *p* = 0.068; ch.7).

Note that, as a result of additional statistical analysis of the effect of the affected side on the beta value change in ch.11, no significant difference was observed between the patients with left paralysis and those with right paralysis [*p* = 0.12; Real group (10 patients with left affected hand vs. 10 patients with right affected hand), *p* = 0.91; Sham group (six patients with left affected hand vs. four patients with right affected hand) Wilcoxon rank-sum test]. Furthermore, it is also necessary to consider the effect of the cognitive fatigue caused by neurofeedback training on beta value changes. However, as mentioned in the Section “2. Materials and methods,” no patients reported severe fatigue during or after training; thus, the effect of fatigue in this study can be assumed to be minimal.

### 3.2. Target-searching task performance

**FIGURE 4:**
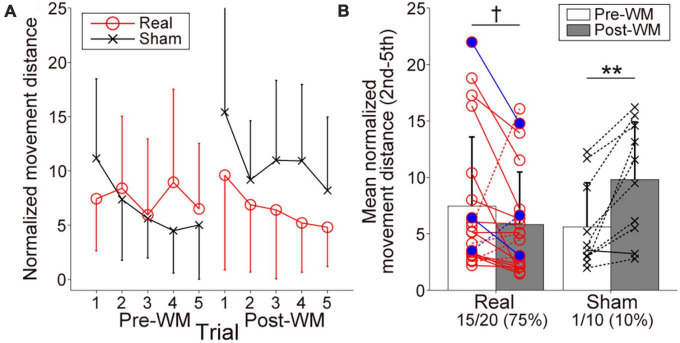
Behavioral performances reflecting working memory (WM) in the target-searching task. **(A)** Normalized movement distance transitions in the Pre- and Post-WM tasks. Red circle lines and black cross lines represent the Real and Sham groups, respectively. In both the Pre- and Post-WM task, patients successfully reduced the normalized movement distance, indicating optimization of the searching movement trajectory. **(B)** Mean normalized movement distance changes in the Real and Sham groups. Red circle lines and black cross lines represent the individual mean normalized movement distance values. Blue-filled circles indicate the patients with mild cognitive dysfunction in the Real group. The solid and dotted lines indicate improvement and worsening trends, respectively. The performance improved in 3/4 of the patients in the Real group, whereas improvement was observed in only one patient in the Sham group. Error bars represent standard deviations. The dagger and asterisks indicate the marginal and significant differences in the normalized movement distance between the Pre- and Post-WM tasks, respectively. ^†^*p* < 0.1, **p < 0.01.

Figure 4A represents the searching performance in the Real and Sham groups. Both groups gradually reduced normalized movement distance from the second to the fifth trials, reflecting spatial information retention. Regarding the change of the mean normalized movement distance shown in Figure 4B, the two-way ANOVA revealed a significant interaction of group × task phase, *F*(1,28) = 13.34, *p* = 0.001, η*_*p*_*^2^ = 0.32, and the main effects did not reach statistical significance, *F*(1,28) = 0.35, *p* = 0.56, η*_*p*_*^2^ = 0.012 for group and *F*(1,28) = 2.61, *p* = 0.12, η*_*p*_*^2^ = 0.085 for task phase. The searching performance in the Sham group was significantly worsened with the Post-WM task (*p* = 0.0032; simple main effect test with the Bonferroni correction). By contrast, although the searching performance in the Real group tended to improve with the Post-WM task compared to the Pre-WM task, the difference was not statistically significant (*p* = 0.089; simple main effect test with the Bonferroni correction). Of note, one patient in the Real group showed a large normalized movement distance increase. When the two-way ANOVA was conducted again after excluding this outlier (i.e., with 19 patients in the Real group and 10 patients in the Sham group), we also found a significant interaction of group × task phase [*F*(1,28) = 22.93, *p* = 0.000054, η*_*p*_*^2^ = 0.46 for interaction, *F*(1,28) = 0.49, *p* = 0.49, η*_*p*_*^2^ = 0.018 for group and *F*(1,28) = 2.08, *p* = 0.16, η*_*p*_*^2^ = 0.071 for task phase]. The searching performance significantly improved in the Real group (*p* = 0.0083; simple main effect test) but significantly worsened in the Sham group (*p* = 0.00066; simple main effect test). Furthermore, the proportion of patients with improved the searching performance (i.e., decreased mean normalized movement distance), corresponded to 75% (15 of 20 patients) in the Real group and 10% (1 out of 10 patients) in the Sham group. In the Real group, two of three patients diagnosed with mild cognitive dysfunction showed the searching performance improvements. In the Sham group, the searching performance of only one patient improved, showing a small improvement. The group difference for the proportion of patients with improved performance was significant (*p* = 0.00077).

### 3.3. Effect of neuromodulation on WM performance

**FIGURE 5:**
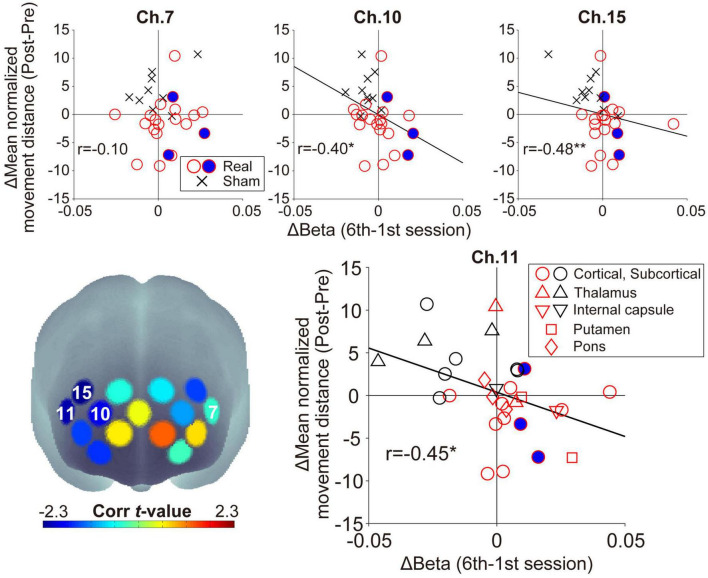
Relationship between the individual beta value changes during neurofeedback training and the normalized movement distance changes from Pre-WM to Post-WM tasks. The lower-left panel shows the spatial configurations of the *t*-values from the correlation analyses. There was a significant negative correlation in ch.11 (right feedback channel), ch.10, and ch.15, but ch.7 (left feedback channel) did not show a significant correlation. The negative correlations indicate that patients achieving higher task-related activity during the neurofeedback training task also demonstrated higher behavioral performance improvement from the Pre-WM task to the Post-WM task. The blue-filled circles indicate the patients with cognitive dysfunction based on the Trail Making Test or the Behavioral Inattention Test. The asterisks indicate a significant correlation between the beta value changes and the normalized movement distance changes. In the lower-right panel showing the distribution of ch.11, the shape is changed according to the individual stroke lesion, and red and black shapes indicate the Real and Sham groups, respectively. *p < 0.05, **p < 0.01.

In the feedback channels, there was a significant negative correlation between the inter-subject variance of the oxy-Hb beta value changes and that of the mean normalized movement distance changes only at ch.11 (ch.7: *r* = −0.10, *p* = 0.59, ch.11: *r* = −0.45, *p* = 0.014; Figure 5). In addition to ch.11, significant negative correlations were found for other right prefrontal areas in ch.10 (*r* = −0.40, *p* = 0.029) and ch.15 (*r* = −0.48, *p* = 0.008). In other words, lower normalized movement distance (i.e., higher WM ability to hold spatial information) was found after neurofeedback training in individuals acquiring higher right prefrontal activities during neurofeedback training. There was no significant correlation for channels other than those shown in Figure 5 (−0.34 < rs < 0.28, *p*s > 0.07). Neurofeedback training success was defined as an increase in prefrontal activity in the neurofeedback channel ch.11 and an improvement in the searching performance (i.e., patients distributed in the fourth quadrant in Figure 5), then 11/20 patients can be considered successfully trained in the Real group. Conversely, no patient in the Sham group showed a successful training trend.

Regarding ch.11, which showed a significant correlation as a neurofeedback channel, no marked lesion-specific tendency was observed (lower-right panel in Figure 5). Furthermore, regarding patients with cognitive dysfunction, all three patients successfully showed higher prefrontal activities corresponding to the current neurofeedback training aims, and two of them also improved their searching performance. No patient-specific trends with cognitive dysfunction, such as showing outliers within the Real group distribution, were observed. Furthermore, there was no significant correlation between the patients’ backgrounds (FMA motor score and time since stroke) and the experimental outcomes (beta value and searching performance) in the Real group (−0.05 < rs < 0.02, *p*s > 0.84).

## 4. Discussion

### 4.1. Effectiveness of short-term neurofeedback training for patients with acute stroke

The present short-term neurofeedback training led to higher activity in the right prefrontal area when applied to acute stroke patients. Furthermore, WM performance did not decline in patients who displayed this task-related prefrontal activity increase. These findings provide evidence that neurofeedback training has positive effects in the acute stroke phase similar to those shown in many previous studies in the chronic phase (Mihara and Miyai, 2016; Renton et al., 2017; Wang et al., 2018). The present training protocol aimed to acquire higher bilateral prefrontal activities (van Asselen et al., 2006; Jones et al., 2015; Stephens and Berryhill, 2016); moreover, the acquisition of higher prefrontal activity contributed to the prevention of the decline in WM performance. Because these trends were clearly observed only in the Real group, we can conclude that a neurofeedback training protocol in which patients with acute stroke monitor their own brain activity is effective in providing neuromodulation and concomitant cognitive improvement.

For individual results, patients in the Real group who acquired higher right prefrontal activity during neurofeedback training showed a greater degree of improvement in WM performance. Regarding the training success rate, over 50% (11 out of 20 patients) of the patients in the Real group achieved both increased brain activity in the right feedback channel and improved WM performance. Although the success rate of our neurofeedback training protocol might seem modest, the present neurofeedback training was as effective as that reported previously (Alkoby et al., 2018). Therefore, also from the training success rate viewpoint, the current results would support short-term neurofeedback training effectiveness for acute stroke patients.

Because cognitive decline after stroke often leads to decreased rehabilitation participation (Skidmore et al., 2010), a neurofeedback training protocol that maintains cognitive function in acute stroke patients might have the potential to encourage rehabilitation in a greater number of patients. Indeed, in a clinical study exploring the time of rehabilitation onset’s effect on functional prognosis, starting rehabilitation on admission day or the following day was more beneficial than starting it 3 days after admission (Momosaki et al., 2016). Although the current findings only revealed the effect for a limited period, i.e., immediately after training, it is implied that the neurofeedback technique also contributes to promoting brain plasticity as early as possible in the acute phase, even in terms of psychological meaning such as motivation.

### 4.2. Role of the right prefrontal cortex

The short-term neurofeedback training successfully contributes to preventing WM performance decline with higher right prefrontal activity. Previous studies have indicated that the right DLPFC is involved in processing different types of visuospatial WM information. For instance, higher activity levels in the right DLPFC induced by anodal transcranial direct current stimulation enhanced the accuracy in memorizing visuospatial locations (Giglia et al., 2014; Wu et al., 2014). In addition, the FPC is involved in visual-spatial memory (Owen et al., 2005; Slotnick and Moo, 2006). Thus, acquiring higher right DLPFC activity, rather than the left one, might reflect the active spatial processing during neurofeedback training tasks. Conversely, the left prefrontal area is important for maintaining internal body information, such as tactile and somatosensory information during cognitive tasks (Pleger et al., 2006; Kaas et al., 2007). However, the present neurofeedback task required only spatial processing based on vision; therefore, patients might have failed to control the left area activity even though they received feedback from this area. Thus, the association between the activity level in the right DLPFC and FPC through neurofeedback training and the WM performance observed in this study is a reasonable result because these regions are directly involved in WM related to spatial processing.

Although increased activity in the right DLPFC can contribute to preventing a decline in WM performance, it should be noted that another risk might also be induced. For instance, there is an association between right DLPFC activity and depression. Previous studies reported that applying low-frequency transcranial magnetic stimulation to the right DLPFC to suppress activity improved depressive symptoms (Stern et al., 2007; Fitzgerald et al., 2009). These results suggest that excessively increasing the activity in the right DLPFC may increase the risk of depression. A short training duration, as used in the current study, might have a low risk. However, when considering future long-term rehabilitation applications, the appropriate amount of training and activity level of the right DLPFC need to be defined.

### 4.3. Influential factor on the neurofeedback training efficacy

To investigate whether neurofeedback training is effective for acute stroke patients, it is also important to consider individual differences in training efficacy. Indeed, recent studies often pointed out that individual neurofeedback training efficacy varies widely (Alkoby et al., 2018; Diaz Hernandez et al., 2018; Kadosh and Staunton, 2019; Sakurada et al., 2022). This individual variability in the neurofeedback training efficacy is likely even greater in clinical populations because of pathological condition influences. Importantly, at least in the current population, no relationship between neurofeedback efficacy and individual clinical backgrounds, such as FMA motor score and the time after stroke onset, was observed. Moreover, it has been confirmed that cognitive dysfunction does not necessarily eliminate the neurofeedback efficacy (two of three patients with mild cognitive dysfunction showed positive training results). Of course, although further investigation based on a larger stroke cohort is warranted to conclude that individual clinical backgrounds do not influence neurofeedback efficacy, neurofeedback training was expected to be effective in a wide range of clinical populations, including acute phase patients with cognitive dysfunction.

Note that, as mentioned above, the neurofeedback training protocol’s success rate was modest. The influential factors on the training efficacy will need to be identified to overcome the individual differences in neurofeedback training efficacy and increase the success rate in stroke patients in the future. In fact, stroke patients’ clinical factors that are important for predicting prognosis and various new predictors will need to be considered as they were identified in recent studies (Abe et al., 2021; Moore et al., 2021). Additionally, neurophysiological factors can relate to the ability to regulate brain activity using neurofeedback. Previous studies have reported a relationship between various brain activity patterns, which reflect individual brain function characteristics and neurofeedback training efficacy. For instance, the power of alpha EEG frequency during the eyes-closed and eyes-open resting states prior to neurofeedback training was identified as significant predictors for successful EEG learning, with the eyes-closed state being more accurate for predicting neurofeedback training efficacy (Wan et al., 2014). Thus, individual original neural dynamics might determine neurofeedback training efficacy. Accumulating evidence about the influential factors would increase neurofeedback training significance as a desirable neurorehabilitation approach.

Regarding the neurofeedback efficacy, no lesion-dependent tendency, such as impairment of specific areas, was observed. If this result is interpreted positively, neurofeedback training can be efficacious in the acute phase regardless of the stroke lesion site. However, we should consider the relationship between the stroke lesion site and the network involved in neurofeedback training efficacy. Focusing on the basal ganglia, the nucleus accumbens is part of the ventral striatum and is related to motivation (Nakamura et al., 2012), receiving inputs from the insular cortex, which is involved in cognitive functions such as emotion and behavior expression (Cauda et al., 2012). Therefore, damage to the nucleus accumbens might hinder motivation for training. For instance, the putamen, a region close to the nucleus accumbens was affected in the current patients. Thus, a lesion in this area might decrease neurofeedback training efficacy due to decreased motivation for training, based on the basal ganglia network. Since the patients in this study completed all sessions, it is unlikely that individual stroke conditions affected the neural basis of training motivation. When conducting neurofeedback training in stroke patients, we need to consider not only the brain areas to be trained but also factors and neural substrates indirectly related to training performance.

### 4.4. Limitations

Regarding the study design, all essential items in the checklist for neurofeedback research (Ros et al., 2020) were satisfied, except for pre-designing the sample size, which was difficult due to the limited study period. Accordingly, a major limitation of this study is the relatively small cohort size. Therefore, the effects of heterogeneous pathologies on the neurofeedback training efficacy could not be examined. Indeed, the number of patients was insufficient to examine the effects of the lesion type or location. Larger-scale studies are needed to address if the stroke type, the lesion type, and the lesion location influence the neurofeedback training efficacy. Furthermore, we currently studied a one-time intervention; therefore, longer neurofeedback training might have additional benefits, and show further associations with other clinical or demographic factors. Finally, in future investigations, it is important to compare the stroke group with and age-matched healthy group, to clarify whether the degree of neurofeedback training efficacy observed in patients with stroke is similar to that of the healthy population.

## 5. Conclusion

We demonstrated that short-term fNIRS-based neurofeedback training can strengthen the right prefrontal activity and prevent WM ability decline, at least immediately after the training. Thus, the current findings imply that neurofeedback training is an effective approach for patients with acute stroke and may be clinically feasible via the combination of neurofeedback training and traditional occupational therapy. In the future, to identify the factors that affect neurofeedback training efficacy, it is necessary to evaluate its relationship with various clinical parameters in a larger population.

## Data availability statement

The raw data supporting the conclusions of this article will be made available by the authors, without undue reservation.

## Ethics statement

The studies involving human participants were reviewed and approved by the Institutional Review Board at Jichi Medical University. The patients/participants provided their written informed consent to participate in this study.

## Author contributions

TS conceived, designed the experiment, developed the experimental system, and wrote the draft of the manuscript. TS and KK supervised the study. MT, TN, MiM, SF, and KK provided resources. MT and MaM performed the participant experiments. MT, TS, and MaM analyzed the data. All authors contributed to the discussion of the results and read and approved the final manuscript.

## Footnotes

1
https://www.nitrc.org/projects/homer2/
