# Supplementary material for: Higher prefrontal activity based on short-term neurofeedback training can prevent working memory decline in acute stroke
Source: Front Syst Neurosci. 2023 Jun 14;17:1130272. doi: 10.3389/fnsys.2023.1130272 (PMC10300420; doi:10.3389/fnsys.2023.1130272)
Supplement: Supplementary file 1 [file Data_Sheet_1.pdf]

Supplementary Material

1 Supplementary Figure

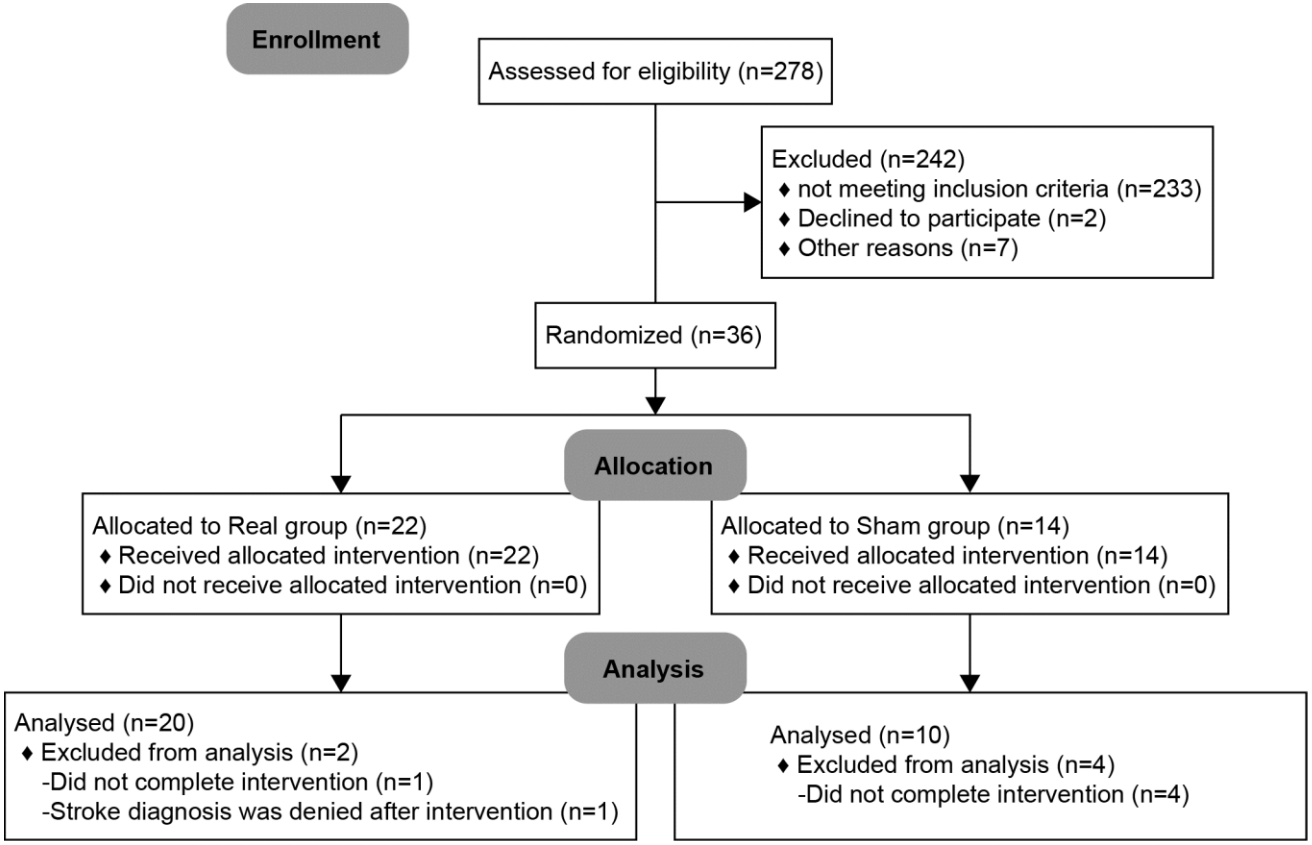

Supplementary Figure 1. CONSORT flow diagram.

2 Supplementary Table

Supplementary Table 1. Participant information. The "+" signs in the column of the MMSE score indicate cognitive dysfunction based on tests other than MMSE performed before the experimental participation. Patient #7 (R06) and participant #17 (R11) scored 395 s and 435 s in the Trail Making Test (Part B), respectively, indicating the possibility of frontal lobe dysfunction. Participant #30 (R20) scored 82 in the Behavioral Inattention Test, indicating partial behavioral neglect. The "–" signs in the column of the MMSE score indicate the absence of cognitive dysfunction based on additional assessment. Patient #20 (R14) scored 86 s and 128 s in the Trail Making Test Part A and B, respectively. Patient #26 (R18) scored 18 in the Frontal Assessment Battery and 74 s and 92 s in the Trail Making Test Part A and B, respectively. Patient #01 (S01) scored 75 s and 84 s in the Trail Making Test Part A and B, respectively. Patient #10 (S04) scored 25 in the revised version of Hasegawa's Dementia Scale. Patient #11 (S05) scored 87 s and 144 s in the Trail Making Test Part A and B, respectively. Patient #29 (S10) scored 67 s and 77 s in the Trail Making Test Part A and B, respectively.

	Age (years)	Gender	Handed-ness	Affected side	Time since stroke (days)	Stroke type	Lesion location	MMSE (/30)	FMA (/66)	MMT (/5)
Real										
R01 (P02)	71	F	Rt	Rt	4	In	Lt corona radiata	28	59	5-
R02 (P03)	36	F	Rt	Lt	13	In	Rt frontal gyrus (SC)	25	59	5-
R03 (P04)	69	M	Rt	Rt	2	In	Lt pons	30	60	5-
R04 (P05)	71	M	Rt	Lt	1	Hem	Rt putamen	29	54	4+
R05 (P06)	73	F	Rt	Rt	3	In	Lt corona radiata	25	47	4
R06 (P07)	78	M	Rt	Rt	5	In	Lt corona radiata	27^{+}	56	5-
R07 (P13)	54	F	Rt	Lt	8	In	Rt pons	30	59	5-
R08 (P14)	67	M	Rt	Lt	10	In	Rt corona radiata	29	58	5-
R09 (P15)	72	M	Rt	Rt	8	In	Lt internal capsule	30	58	5-
R10 (P16)	55	F	Rt	Rt	2	Hem	Lt pons	29	51	4+
R11 (P17)	69	M	Rt	Lt	7	In	Rt front-temporal gyrus (SC)	28^{+}	53	4+
R12 (P18)	78	F	Rt	Lt	5	In	Rt thalamus	28	52	5-
R13 (P19)	69	F	Rt	Rt	4	In	Lt corona radiata	27	52	5-
R14 (P20)	42	M	Rt	Lt	3	Hem	Rt putamen	28^{-}	55	4+
R15 (P21)	72	F	Rt	Lt	6	In	Rt corona radiata	29	53	4+
R16 (P22)	42	F	Rt	Rt	5	Hem	Lt thalamus	26	57	5-
R17 (P25)	66	M	Rt	Rt	10	In	Lt frontal gyrus (SC)	28	53	5-
R18 (P26)	36	F	Rt	Rt	7	Hem	Lt corona radiata	30^{-}	51	4+

	Age (years)	Gender	Handed-ness	Affected side	Time since stroke (days)	Stroke type	Lesion location	MMSE (/30)	FMA (/66)	MMT (/5)
R19 (P28)	43	M	Rt	Lt	7	In	Rt corona radiata	27	55	5-
R20 (P30)	73	M	Rt	Lt	3	Hem	Rt. parietal gyrus (SC)	25^{+}	51	4+
Sham										
S01 (P01)	64	M	Rt	Lt	12	Hem	Rt frontal gyrus (SC)	29^{-}	56	5-
S02 (P08)	52	M	Rt	Rt	2	In	Lt thalamus	28	57	5-
S03 (P09)	68	F	Rt	Lt	1	In	Rt thalamus	27	56	5-
S04 (P10)	68	F	Rt	Rt	9	In	Lt thalamus	25^{-}	56	5-
S05 (P11)	76	M	Rt	Rt	4	In	Lt corona radiata	29^{-}	55	5-
S06 (P12)	55	M	Rt	Lt	3	In	Rt internal capsule	28	53	5-
S07 (P23)	68	F	Rt	Rt	4	In	Lt frontal gyrus (SC)	29	60	5-
S08 (P24)	64	M	Rt	Lt	10	In	Rt frontal gyrus (SC)	26	55	5-
S09 (P27)	77	F	Rt	Lt	9	In	Rt corona radiata	27	57	5-
S10 (P29)	49	M	Rt	Lt	3	Hem	Rt corona radiata	29^{-}	53	4

MMSE: Mini-Mental State Examination, FMA: Fugl–Meyer Assessment, MMT: Manual Muscle Test, F: Female, M: Male, Lt: Left, Rt: Right, In: Infarction, Hem: Hemorrhage, SC: Subcortical cerebrum
